# S12. THE ‘AUTOANTIBODYOME’ IN PSYCHOSIS: A PILOT STUDY AND BLUEPRINT FOR BIOMARKER DISCOVERY

**DOI:** 10.1093/schbul/sby018.799

**Published:** 2018-04-01

**Authors:** Thomas Pollak, Cassandra DeMarshall, Abhirup Sarkar, Timothy Nicholson, Philip McGuire, James Stone, Anthony David, Robin Murray, Robert Nagele

**Affiliations:** 1 Institute of Psychiatry, Psychology & Neuroscience, King’s College London; 2 Rowan University School of Osteopathic Medicine

## Abstract

**Background:**

Recent studies seeking to describe the prevalence and significance of autoantibodies in psychotic disorders can be characterized as ‘top-down’ in theoretical approach; that is, autoantibodies to specific (usually CNS) antigens are sought based on a) the known function of the antigen (e.g. NMDAR) and its putative role in psychosis or b) the clinical observation that these autoantibodies can cause psychosis as part of a more complex neuropsychiatric presentation (e.g. autoimmune encephalitis). No candidate autoantibodies with a clear diagnostic or prognostic role have been definitively established.

We sought to take an alternative, ‘bottom-up’ approach to the significance of autoantibodies in psychosis that remains agnostic as to the potential significance of any one antigen. Every individual harbours autoantibodies directed against many thousands of self-antigens and the vast majority are not disease-causing. Indeed production of autoantibodies may represent a physiological, antigen-specific ‘debris-clearing’ response to tissue destruction or damage. It follows that the autoantibody profile of any individual reflects any pathological process that is ongoing in that individual and can thus serve as a ‘readout’ of the disease state in question.

**Methods:**

Sera from 20 patients with a first episode of psychosis (FEP) (males: n=16; mean age: 29.35 s.d.: 7.07) from the Genetics and Psychosis (GAP) study and 20 matched controls were analysed, using Invitrogen’s ProtoArray v5.1 Human Protein Microarrays, for the presence of IgG to 9486 unique human protein antigens which had been expressed as GST fusion proteins in insect cells, purified and spotted onto slides. Following application of a fluorescent secondary IgG, reactivity patterns were automatically read using a fluorescence scanner. Samples were split into testing and training sets, and the top 50 most differentially expressed and differentially depleted antibodies were then chosen as biomarkers.

**Results:**

The top 50 expressed biomarkers from the training set correctly identified 100% of psychosis subjects from the testing set, and 80% of healthy controls (OOB estimate of error rate 10%). When training and testing sets were swapped, biomarker overlap was 46% and 90% of psychosis subjects and 90% of controls were correctly identified (OOB estimate of error rate 10%). The top 50 depleted biomarkers from the training set correctly identified 90% of psychosis subjects from the testing set, and 90% of healthy controls (OOB estimate of error rate 10%). When training and testing sets were swapped, depletion biomarker overlap was 2% and 70% of psychosis subjects and 40% of controls were correctly identified (OOB estimate of error rate 45%).

**Discussion:**

The autoantibodyome in FEP differs from that of healthy individuals. In this novel pilot study, a panel of 50 differentially expressed autoantibodies allowed confident discrimination between patients and controls, potentially paving the way for development of antibody-based diagnostics for psychosis using a simple blood test and fewer autoantibodies. Depletion biomarkers, thought to represent antibodies selectively depleted from the blood due to target binding in tissues, had less replicability and utility than expression biomarkers, which may offer insights into the active role of the adaptive immune system in psychosis. Further work will attempt to validate this approach in larger samples, using psychiatric disease controls. This autoantibodyomic approach may also show promise for the identification of predictive and prognostic biomarkers in psychotic disorders.

